# Comparative histomorphometrical study of genital tract in adult laying hen and duck

**Published:** 2012

**Authors:** Ahmad-Ali Mohammadpour, Abdolkarim Zamanimoghadam, Massoumeh Heidari

**Affiliations:** 1 *Department of Basic Sciences, Faculty of Veterinary Medicine, Ferdowsi University of Mashhad, Mashhad, Iran; *; 2 *Department of Clinical Sciences, Faculty of Veterinary Medicine, Shahrekord University,Shahrekord, Iran; *; 3 *Graduated from Faculty of Veterinary Medicine, Shahrekord University,Shahrekord, Iran.*

**Keywords:** Histomorphometry, Genital tract, Hen, Duck

## Abstract

This study was carried out to compare the histomorphological structures of oviductal regions of the apparently healthy adult laying hens (*Gallus gallus dometicus*) and ducks (*Ansa ansa domesticus*). For this purpose, 20 hens and 20 female ducks aged between 1–1.5 years were used. After euthanasia, the oviducts were dissected out and all of the gross morphometrical parameters including length, width and thickness as well as weight and length of them were recorded. For histological studies, after tissue preparation and staining with H&E, histological layers of isthmus, uterus and vagina were recognized and the size of them with micrometry method were determined. Our data analyses indicated that, the mean weight, length of oviduct as well as weight of isthmus, uterus in hen were significantly (*P *< 0.05) greater than that of duck, whereas the vaginal thickness and weight were greater in duck than the hen. In histological studies, epithelium and cilia were well developed in duck and lamina propria was filled with glands in the regions of the isthmus and uterus. The length of primary mucosal folds of isthmus and uterus in duck was more than hen. The longest mucosal fold has been seen in uterus. Most of the parameters in duck were greater than hen except the length of secondary fold of three parts of oviduct including isthmus, uterus, and vagina.

## Introduction

The avian oviduct is a complex biological organ that undergoes a series of hormonal, neural, biochemical and cellular changes during the formation of an egg. It is of special interest to the commercial egg industry. Any alteration or deviation in the function of the oviduct of a laying hen can directly affect egg and egg shell quality. Decline in egg and egg shell quality cost the egg industry millions of dollars every year. The avian oviduct is divided into five regions, namely infundibulum, magnum, isthmus, uterus or tubular shell gland and vagina. The infundibulum forms a strong perivitelline membrane and chalaza around the egg yolk, the magnum is responsible for the synthesis and secretion of albumen, the isthmus forms a fibrous membrane around the egg white, the uterus forms the egg shell and finally the vagina connects the uterus to the cloaca. The tubular shell gland was initially known as the red region of the isthmus, but because of its role in egg shell formation, it was later named the tubular shell gland.^1^ In all of the oviduct regions, epithelium of the tunica mucosa is composed of ciliated and non-ciliated secretory cells. The lamina propria is filled with compactly arranged glands whose properties vary between the regions of the magnum, isthmus and uterus. The tunica mucosa forms convolutions which protrude into the lumen. These mucosal convolutions which bulge towards the lumen in the form of primary, secondary and tertiary branches are lined with mostly ciliated cells along the luminal surface are mainly composed of secretory cells in the basal region.^2^^-^^4^ The avian oviduct has been studied extensively in some poultry birds, specially the domestic fowl.^5^^-^^9^ The general morphology and overall function of the avian oviduct, especially in *Gallus domesticus,* have been studied for many years.^10^^-^^12^ The information on histomorphology of avian oviduct is still incomplete. 

In the present study, histomorphological structures of genital tract in adult laying duck and hen were compared.

## Materials and Methods

Forty adult and apparently healthy ducks and hens (each comprised of 20 birds) aged between 1–1.5 years were utilized in this investigation. They were obtained from local natural population near Shahrekord, Iran. They were killed by cervical dislocation according to ethical committee of Shahrekord University. Then, whole oviduct was quickly dissected out and stretched on a paper. After dissection, some morphological parameters such as total weight and length of oviduct were measured and then in each of specimen length, width and thickness of three parts of oviduct were measured by caliper device; an instrument used for measuring round and irregular shape objects. For histological studies, tissue samples were obtained from the midpoint of three part of oviduct (isthmus, uterus and vagina) and fixed in 10% buffered formalin and processed for routine microtome. After tissue preparation and H&E staining, histological layers of isthmus, uterus and vagina such as tunica mucosa, submucosa and muscularis were recognized and their dimensions, primary and secondary folds of tunica mucosa with micrometry method were measured. The data were statistically analyzed with Student *t*-test using SigmaStat (version 2.0) statistical software. 

## Results

Morphological examination revealed that the oviduct of laying hen and duck is a highly convoluted and muscular part, which transport ovum from ovary, the place for fertilization, deposition of albumen, formation of egg membranes and finally to form the full-grown egg. It extends from the single ovary to cloaca and occupying a large part of the abdominal cavity. In hen, mean weight (56.15 ± 9.67 g) and mean length (71.85 ± 5.45 cm) of oviduct were significantly (*P *< 0.05) bigger than duck (Table 1). In laying hen, like other parts of oviduct the size and width of uterus were increased. The isthmus is the region where the egg shell membrane is formed and its wall was thinner than that of the uterus and vagina.

Also in hen, the weight (5.76 ± 1.84 g), width (12.33 ± 1.40 mm) and length (14.05 ± 4.34 cm) of isthmus were greater than duck. These parameters in duck were 5.30 ± 3.05 g, 12.18 ± 3.36 cm and 8.92 ± 1.65 mm, respectively. The uterus appears as sac-like dilatation present between the isthmus cranially and the vagina caudally. The wall of the uterus was thicker than that of the isthmus in both birds. In duck, the uterus was lighter and its length was shorter than hen and the differences were significant (*P* < 0.05). Uterus weight in hen (14.98 ± 2.54 g) was more than duck (9.27 ± 2.05 g) and the difference was significant (*P* < 0.05). Other parameters such as width of uterus in hen were more than duck but thickness of uterus in duck was more than hen. There was no significant difference in length of uterus between hen and duck.

The vagina was tube-like structure connected to the uterus cranially and opened on the urodeum of the cloaca caudally. The vaginal wall was thicker than that of the other portions of the oviduct. Most of parameters in vaginal part of duck's oviduct were more than of hen's oviduct. Its wall thickness in duck was more than hen. It was recorded 2.01 ± 0.78 mm and 3.31 ± 1.11 mm in hen and duck, respectively. There was significant difference in vaginal wall thickness between duck and hen (*P* < 0.05) (Table 1).

**Table 1:** Different parameters of the genital organs in duck and hen (Mean ± SD).

**Parameters**	**Hen**	**Duck**
**Total weight of oviduct (g)**	56.15 ± 9.67*	35.69 ± 9.90
**Total Length of oviduct (cm)**	71.85 ± 5.45*	60.27 ± 10.19
**Isthmus weight (g)**	5.76 ± 1.84	5.30 ± 3.05
**Isthmus length (cm)**	14.05 ± 4.34	12.18 ± 3.36
**Isthmus width (mm)**	12.33 ± 1.40*	8.92 ± 1.65
**Isthmus thickness (mm)**	1.60 ± 0.45	1.64 ± 0.29
**Uterus weight (g)**	14.98 ± 2.54*	9.27 ± 2.05
**Uterus length (cm)**	7.70 ± 0.82	7.27 ± 1.08
**Uterus width (cm)**	5.15 ± 0.70*	3.37 ± 0.88
**Uterus thickness (mm)**	2.07 ± 0.61	3.44 ± 1.41*
**Vagina weight (g)**	3.85 ± 1.05	8.41 ± 1.38*
**Vagina length (cm)**	6.10 ± 2.10	6.62 ± 1.13
**Vagina width (mm)**	11.65 ± 4.18	14.85 ± 2.78
**Vagina thickness (mm)**	2.01 ± 0.78	3.31 ± 1.11*

* indicates significant differences, *P *< 0.05.

**Fig. 1:**
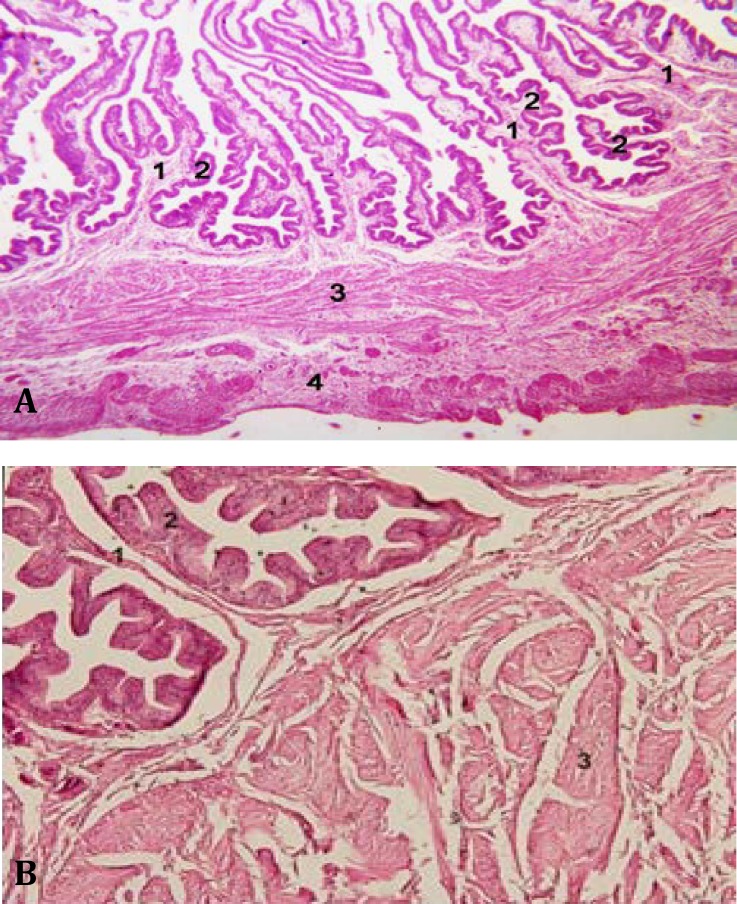
Histology of vagina in hen (A) and duck (B).Primary fold (1). Secondary fold (2). Tunica muscularis (3). Tunica serosa (4) (H&E, (**A**: 64×, **B**: 160×).

In histological observations the oviducts were divided into five regions in both species, namely tunica mucosa, tunica muscularis and tunica serosa. The tunica mucosa was to form convolutions protruding into the lumen, and the epithelium was composed of pseudo-stratified columnar with ciliated and non-ciliated secretory cells.

**Fig. 2:**
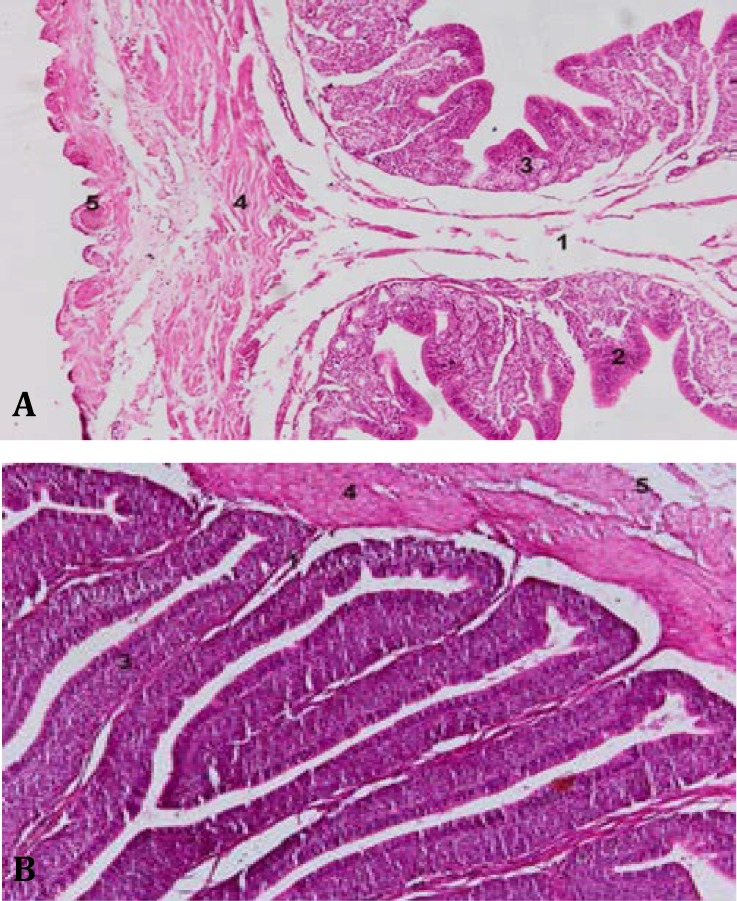
Histology of Isthmus in hen (A) and duck (B). Primary fold (1). Secondary fold (2). Lamina propria and glands(3). Tunica muscularis (4). Tunica serosa (5). (H&E.** A**: 160×, **B**: 320×).

In laying duck, the epithelium and cilia were to be well developed and lamina propria was filled with glands in the regions of the isthmus and uterus. The vagina did not have any glands in the lamina propria (Figs. 1 and 2). In histomorphometrical studies, the length of mucosal folds of isthmus and uterus in duck was more than duck. The longest mucosal fold was seen in uterus. It was observed 1337.38 ± 301.90 µm and 1497.27 ± 108.78 µm in hen and duck, respectively. In vagina, tunica muscularis was thicker than other two region of oviduct. It was seen 471.11 ± 204.96 µm and 323.88 ± 67.57 µm in hen and duck, respectively (Table 2).

The muscularis is smooth muscle with inner circular and outer longitudinal layers increasing gradually in thickness. In vaginal wall it was greater than other parts of oviduct. The serosa layer is made up of loose connective tissue covered with mesothelium. In birds just before oviposition, in oviduct there is most advanced caudal part, where the heights and widths of folds of tunica mucosa are more than in cranial part, that probably, is caused for the future function of these departments. During the ovi-position, the tunica mucosa is most advanced in such departments, as: magnum, isthmus, and shell-gland.^13^^,^^14^

**Table 2:** Different histometrical parameters of the genital organs in duck and hen (Mean ± SD).

**Parameters**	**Oviduct region**		
	**Vagina**	**Uterus**	**Isthmus**
**Primary fold length (** **µm)**			
**Hen**	1146.23 ± 156.01	1337.38 ± 301.90	811.44 ± 223.22*
**Duck**	1318.81 ± 244.90*	1497.27 ± 108.78*	658.33 ± 138.79
**Secondary fold length (µm)**			
**Hen**	224.16 ± 80.19	164.47 ± 34.16	119.13 ± 17.73
**Duck**	147.31 ± 65.19	119.80 ± 27.89	106.66 ± 7.47
**Primary fold width (µm)**			
**Hen**	766.11 ± 206.51	1368.05 ± 515.62	905.55 ± 215.05
**Duck**	1181.11 ± 258.43*	1709.72 ± 421.05*	840.55 ± 42.90
**Tunica mucosa width (µm)**			
**Hen**	75.41 ± 13.43	122.22 ± 20.84	337.22 ± 109.467
**Duck**	80.97 ± 36.07	155.20 ± 50.05	448.05 ± 98.40*
**Tunica submucosa width (µm)**			
**Hen**	75.41 ± 30.33	99.58 ± 51.54	154.16 ± 29.76
**Duck**	74.16 ± 38.72	62.22 ± 29.07	185.83 ± 24.63*
**Tunica muscularis width (µm)**			
**Hen**	62.50 ± 21.81	92.77 ± 32.88	471.11 ± 204.96*
**Duck**	81.11 ± 25.64	89.86 ± 67.45	323.88 ± 67.57

* indicates significant differences, *P *< 0.05.

## Discussion

In hen and duck the functional left oviduct consists of five regions: infundibulum, magnum, isthmus, uterus or shell gland and vagina. This organ is fully developed in most adult birds only in left side. Although each of these divisions has structural and functional differences, they all have two morphologic features, essential for egg formation, in common namely (1) a muscular layer, which supports the oviduct and propels the egg and (2) a glandular epithelial lining, which secretes all the parts of the egg outside the yolk-filled oocyte/zygote. Moreover, the mucous membrane produce a slimy secretion that forms a soft resilient cushion for the egg as it passes through the oviduct. The mucosal layer of oviduct wall is made up of ciliated and non-ciliated epithelium with secretory cells. It varies from ciliated columnar to pseudo-stratified columnar in different region of oviduct. In isthmus, the epithelium was in the form of ciliated simple columnar, whereas in the uterus and vagina it was ciliated pseudo-stratified columnar form. The longitudinal oriented folds in the mucosa were extended spirally down the length of the oviduct with variations in their heights and thicknesses.

Structures of oviduct were studied in Punjab white quails. The lining epithelium of infundibulum, magnum, isthmus was simple columnar ciliated with ciliated and non-ciliated cells. The uterus and vagina was lined pseudo-stratified columnar epithelium having ciliated, non-ciliated, and basal and goblet cells. The proprial glands were mainly present in the caudal part of infundibulum, magnum, isthmus and uterus.

Bansal and colleagues in 2010 has reported that the length and number of mucosal folds were maximum in the cranial part of infundibulum whereas, the tunica muscularis was the thickest in the uterus and vagina. The tunica serosa was a thin connective tissue layer covered by mesothelium.^15^ The results of this study have been shown that, in ostrich, while the isthmus was as a short tube with thicker wall the magnum was successive. It has also a well-developed muscular coat than that of the magnum. The luminal surface was folded and the epithelium formed by pseudo-stratified ciliated columnar epithelium, along with some non-ciliated columnar secretory ones. In uterus, luminal surface exhibited series of tall longitudinal mucosal folds with epithelium similar to previous segments. According to Czareva, the epithelium of the glandular secretory tubules were shown a negative reaction to alcian blue stain.^6^

The vaginal wall was thicker than that of the other oviduct portions. The luminal surface of the vagina was composed of the thin longitudinally oriented mucosal folds. The lamina propria was formed of fibrous connective tissue with blood vessels and nerves along with aggregated lymph nodules in the submucosa.^16^

According to the results of the present study, we can concluded that, the gross morphology, histology and histomorphometry of oviduct and its different regions are varies in tested animals. In the laying hen and duck the size and width of uterus were larger than other parts.

## References

[1] Chousalkar KK, Roberts JR (2008). Ultrastructural changes in the oviduct of the laying hen during the laying cycle. Cell Tissue Res.

[2] Balachandran A, Bhatnagar MK, Geissinger HD (1985). Scanning and transmission electron microscopic studies on the oviduct of pekin ducks fed methyl mercury containing diets. Scanning Microsc.

[3] Bakst MR (1998). Structure of the avian oviduct with emphasis on sperm storage in poultry. J Exp Zool.

[4] Ozen A, Ergun E, Kurum A (2009). Light and electron microscopic studies on the oviduct epithelium of pekin duck (Ansa playtrhynchos). Ankara Univ Vet Fak Derg.

[5] Manukhina AI, Stoljarova AG, Donchenko NP (1983). Ultrastructure of oviduct and some endocrine glands at the layer during compulsory moult. VNII Physiol Biochem Feed Anim.

[6] Czareva OY (1990). Features of morphology and histochemistry gland of a tunica mucosa of various departments of oviduct of the hens. Omsk.

[7] Stepina OY (1997). Features of micromorphology and histochemistry of oviduct of the hens after the termination oviposition. Troiczk.

[8] Yoshimura Y, Ogawa H (1998). Histological characterization of the oviducal structures in guinea fowl (Numida meleagris). Japan Poultry Sci.

[9] Mohammadpour AA, Keshtmandi M (2008). Histomorphological study of infundibulum and magnum in turkey and pigeon. World J Zool.

[10] Fuyii S (1981). Scanning electron microscopic observation on ciliated cells of the chicken oviduct in various functional stages. J Fac Appl Biol Sci Hiroshima Univ.

[11] Morris TR (1990). The manipulation of egg size and egg quality. S Afr J Anim Sci.

[12] Aupov FG (1987). Morphological features of synthesis of a shell egg at the layer. Bulletin viii Physiol Biochem Feed Anim.

[13] Khokhlov RY, Kuznetcov SI (2007). Morphogenesis of a Tunica Mucosa of Oviduct of the Hens. Int J MorphoL.

[14] Khokhlov RY (2008). Morphology of an Infundibulum of the Oviduct of the Sexually Mature Hens. Int J Morphol.

[15] Bansal N, Uppal V, Pathak D (2010). Histomorphometrical and histochemical studies on the oviduct of Punjab white quails. Indian J Poultry Sci.

[16] Saber AS, Emara SAM, AboSaeda OMM (2009). Light, Scanning and Transmission Electron Microscopical Study on the Oviduct of the Ostrich (Struthio camelus). J Vet Anat.

